# 5-Aminolevrinic Acid Exhibits Dual Effects on Stemness in Human Sarcoma Cell Lines under Dark Conditions

**DOI:** 10.3390/ijms24076189

**Published:** 2023-03-24

**Authors:** Shohei Horii, Shiori Mori, Ruiko Ogata, Shota Nukaga, Ryoichi Nishida, Shingo Kishi, Rika Sasaki, Ayaka Ikemoto, Takuya Owari, Fumisato Maesaka, Kanya Honoki, Makito Miyake, Yasuhito Tanaka, Kiyohide Fujimoto, Rina Fujiwara-Tani, Hiroki Kuniyasu

**Affiliations:** 1 Department of Molecular Pathology, Nara Medical University, Kashihara 634-8521, Nara, Japan; 2 Department of Urology, Nara Medical University, Kashihara 634-8522, Nara, Japan; 3 Department of Orthopedic Surgery, Nara Medical University, Kashihara 634-8522, Nara, Japan

**Keywords:** ALA, HO-1, carbon monoxide, sarcoma, stemness

## Abstract

5-aminolevulinic acid (ALA) is used for tumor-targeting phototherapy because it is converted to protoporphyrin IX (PPIX) upon excitation and induces phototoxicity. However, the effect of ALA on malignant cells under unexcited conditions is unclear. This information is essential when administering ALA systemically. We used sarcoma cell lines that usually arise deep in the body and are rarely exposed to light to examine the effects of ALA treatment under light (daylight lamp irradiation) and dark (dark room) conditions. ALA-treated human SW872 liposarcoma cells and human MG63 osteosarcoma cells cultured under light exhibited growth suppression and increased oxidative stress, while cells cultured in the dark showed no change. However, sphere-forming ability increased in the dark, and the expression of stem-cell-related genes was induced in dark, but not light, conditions. ALA administration increased heme oxygenase 1 (HO-1) expression in both cell types; when carbon monoxide (CO), a metabolite of HO-1, was administered to sarcoma cells via carbon-monoxide-releasing molecule 2 (CORM2), it enhanced sphere-forming ability. We also compared the concentration of biliverdin (BVD) (a co-product of HO-1 activity alongside CO) with sphere-forming ability when HO-1 activity was inhibited using ZnPPIX in the dark. Both cell types showed a peak in sphere-forming ability at 60–80 μM BVD. Furthermore, a cell death inhibitor assay revealed that the HO-1-induced suppression of sphere formation was rescued by apoptosis or ferroptosis inhibitors. These findings suggest that in the absence of excitation, ALA promotes HO-1 expression and enhances the stemness of sarcoma cells, although excessive HO-1 upregulation induces apoptosis and ferroptosis. Our data indicate that systemic ALA administration induces both enhanced stemness and cell death in malignant cells located in dark environments deep in the body and highlight the need to pay attention to drug delivery and ALA concentrations during phototherapy.

## 1. Introduction

The photoactivation of 5-aminolevulinic acid (ALA) enhances the production of oxidative stress by its metabolite, protoporphyrin IX (PPIX), resulting in antitumor effects [1]. This method has been applied in the treatment of many cancers, including bladder cancer [2]. When administered systemically, 5-ALA is selectively taken up by tumors and converted to PPIX and phototoxic to produce an antitumor effect [2,3]. However, cancer cells that reside deep within the body, such as metastasized cancer cells, are not exposed to light and do not cause phototoxicity from ALA. However, the precise effect of ALA on tumors in the absence of photoactivation is unclear.

Heme oxygenase-1 (HO-1), an inducible form of HO, metabolizes the heme group to biliverdin (BVD), producing Fe^2+^ and carbon monoxide (CO) as co-products [4]. HO-1 is overexpressed in many cancers, and its antioxidant action promotes resistance to oxidative stress in cancer cells, contributing to their survival [5,6]. Thus, HO-1 is involved in promoting malignant traits, such as cancer metastasis [7,8]. Suppression of HO-1 induces prominent cell death in cancer cells [9]. HO-1 is an enzyme in the ALA metabolic system that affects the antitumor activity of ALA under light conditions. However, the effects of HO-1 in response to ALA in the absence of photoactivation remain unclear. Furthermore, HO-1 promotes carcinogenesis, tumor progression, and chemoresistance [10] and is associated with the maintenance and differentiation of normal stem cells [11]. Its relationship with stemness has attracted attention. Stemness is a stem cell property, and in cancer, it is a biological characteristic of cancer stem cells. On the other hand, it has become clear that the activity of HO-1 has both tumor suppression and promotion effects [12].

Sarcomas occur deep inside the body, in locations such as soft tissues and bones, and they exhibit highly malignant phenotypes, such as high metastatic potential and drug resistance [13]. Most sarcomas affect adolescents and young adults, with a 5-year survival rate of about 70–90%, but less than 20% for metastatic cases, and even survivors suffer significant lifelong physical impairment [14,15]. The discovery of new molecular targets can have a great impact on the treatment of sarcoma. In recent years, it has become clear that the stemness of malignant cells is a key cause of metastasis, recurrence, and treatment resistance, and this has been emphasized in sarcomas [16,17]. As sarcomas develop deep inside the body without exposure to light, the application of phototherapy to sarcomas has also been attempted, but deep light irradiation requires technical intervention [18]. We then considered this cancer type to be a suitable model for investigating the effects of ALA under dark conditions. In this study, we investigated the effects of ALA on various hallmarks of cancer, particularly stemness, under dark conditions in human sarcoma cell lines.

## 2. Results

### 2.1. Effect of ALA without Light on Proliferation and Oxidative Stress in Sarcoma Cells

**Figure 1:**
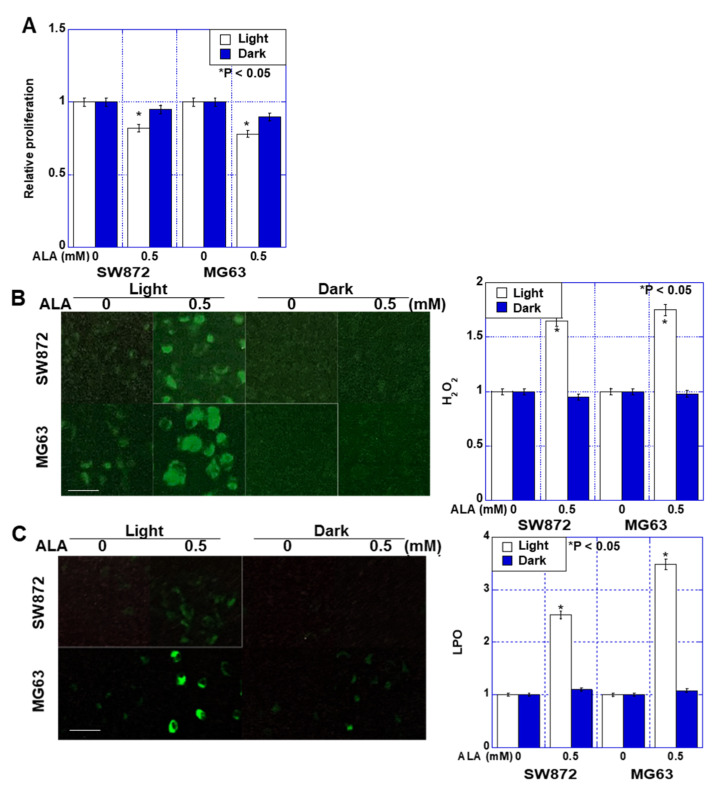
Effect of ALA on cell growth and ROS production in human sarcoma cell lines. Human liposarcoma SW872 and human osteosarcoma MG63 cells were treated with ALA under light (daylight lamp) or dark (dark room) conditions for 48 h. (**A**) Cell growth was evaluated by MTS assay. (**B**,**C**) ROS production was examined by (**B**) DHR123 (mitochondrial H_2_O_2_), scale bar, 20 μm, and (**C**) Liperfluo (mitochondrial LPO), scale bar, 50 μm. The panels on the right show semi-quantified fluorescence intensities. Data in panels, mean ± SD. ALA, 5-aminolevulinic acid; ROS, reactive oxygen species; LPO, lipid peroxide.

We first examined the effects of ALA treatment on the proliferation of liposarcoma SW872 and osteosarcoma MG63 cells (Figure 1A). When both cell lines were exposed to a daylight lamp (light wavelength 400–800 nm), ALA inhibited growth by approximately 20%. In contrast, no significant growth inhibition was observed when ALA treatment occurred in a dark room (dark). Next, we examined the effects of ALA treatment on oxidative stress (Figure 1B,C). Both H_2_O_2_ and 4-HNE increased 1.5–3.5-times under light but did not increase in the dark.

### 2.2. Effect of ALA without Light on Mitochondria in Sarcoma Cells

**Figure 2:**
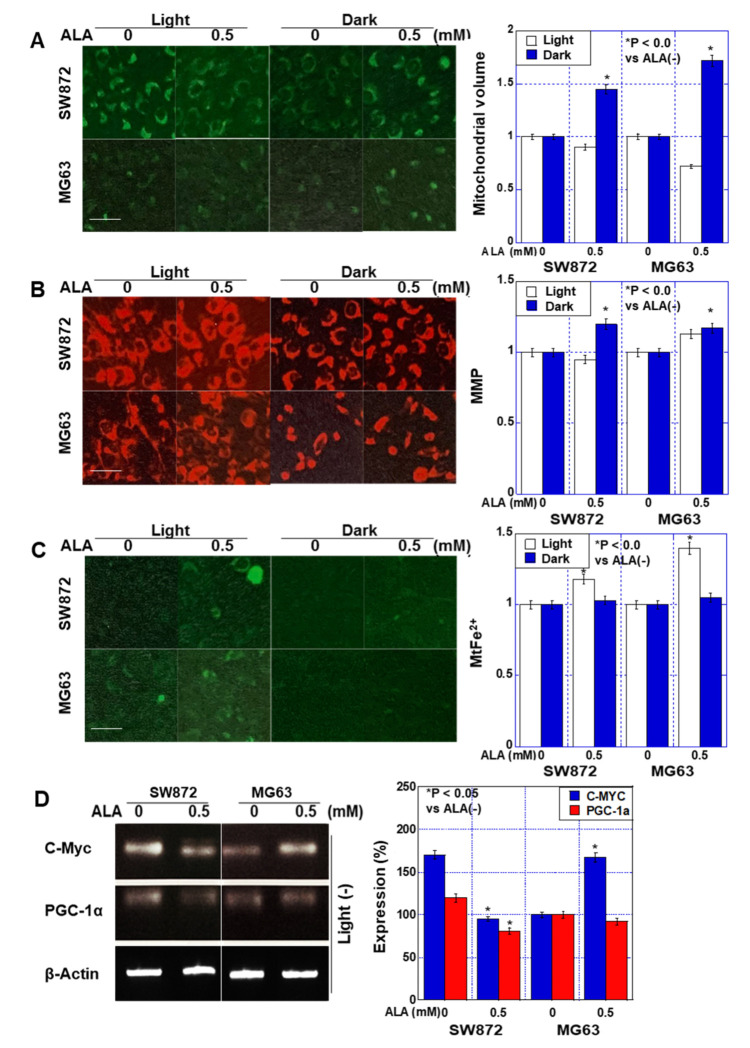
Effect of ALA on mitochondrial properties in human sarcoma cell lines. Human liposarcoma SW872 and human osteosarcoma MG63 cells were treated with ALA under light (daylight lamp) or dark (dark room) conditions for 48 h. (**A**–**C**) Mitochondrial volume was detected by MitoGreen (**A**), MMP was detected by TMRE (**B**), and mitochondrial Fe^2+^ was detected by Mitoferrogreen (**C**). Scale bar, 20 μM. The panels on the right show semi-quantified fluorescence intensities. (**D**) mRNA expression levels of c-Myc (glycolysis) and PGC-1α (oxidative phosphorylation) in dark conditions. The right panel shows the semi-quantification of the RT-PCR signals. Data in panels, mean ± SD. ALA, 5-aminolevulinic acid; MMP, mitochondrial membrane potential; TMRE, tetramethylrhodamine ethyl ester; PGC, peroxisome proliferator-activated receptor-gamma coactivator.

Next, we examined alterations in mitochondria following ALA treatment (Figure 2). ALA treatment increased mitochondrial volume by 45–73% in the dark, whereas it decreased under exposure to light (Figure 2A). The mitochondrial membrane potential was unchanged under light but increased by 20–23% in the dark (Figure 2B). Furthermore, mitochondrial Fe^2+^ increased under light conditions but did not change under dark conditions (Figure 2C). We also examined the effects of ALA on the expression of c-Myc (glycolysis) and peroxisome proliferator-activated receptor-gamma coactivator (PGC)-1α (oxidative phosphorylation), which regulate energy metabolism, in the dark (Figure 2D). c-Myc expression was decreased by ALA treatment in SW872 cells but increased in MG63 cells. In contrast, PGC-1α expression was decreased in both cell types.

These data suggest that under light conditions, ALA induces cell death by increasing oxidative stress and mitochondrial Fe^2+^, and that ALA-induced PPIX was also produced by daylight lamp excitation. Under dark conditions, ALA affected mitochondria but did not induce obvious changes in proliferation or oxidative stress.

### 2.3. Effect of ALA without Light on Stemness in Sarcoma Cells

**Figure 3:**
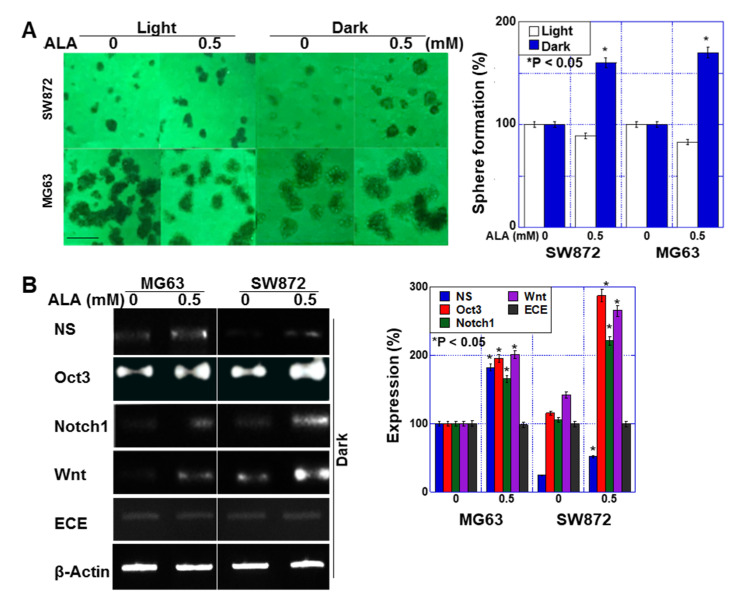
Effect of ALA on stemness in human sarcoma cell lines. Human liposarcoma cell line SW872 and human osteosarcoma cell line MG63 were treated with ALA under light (daylight lamp) or dark (dark room) for 48 h. (**A**) Sphere formation of sarcoma cells. Scale bar, 100 μm. The right-hand panel shows the number of spheres. (**B**) mRNA expression of stemness-related genes as detected by RT-PCR. The right panel shows the semi-quantification of the RT-PCR signals. Data in panels, mean ± SD. ALA, 5-aminolevulinic acid; NS, nucleostemin; Oct3, octamer-binding transcription factor-3; ECE, endothelin-converting enzyme.

Next, we examined the effect of ALA treatment on stemness under dark conditions (Figure 3). The sphere-forming ability of the two sarcoma cell lines was decreased by 10–18% by ALA treatment under light, whereas it was increased by 58–71% in the dark (Figure 3A). When we examined the effect of ALA on the expression of stem-cell-related genes under dark conditions, we observed an increase in the expression of Oct3, nucleostemin (NS), Notch1, and Wnt, but the expression of endothelin-converting enzyme (ECE) was not altered (Figure 3B).

### 2.4. Effects of HO-1 on Stemness in Sarcoma Cells

**Figure 4:**
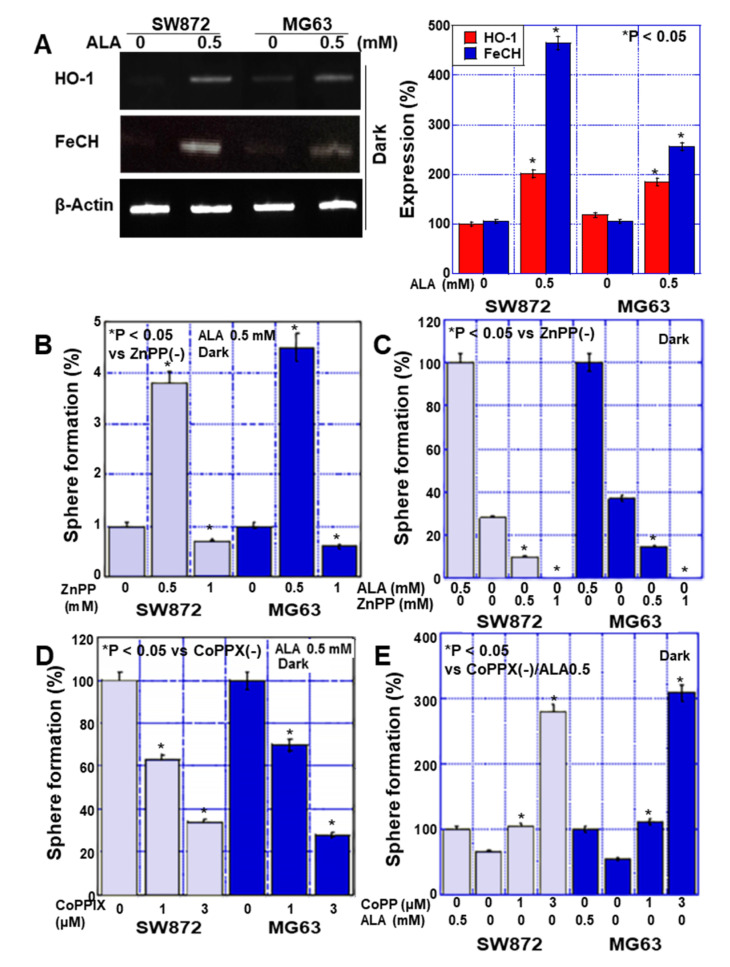
Effect of ALA on sphere formation through HO-1 in human sarcoma cell lines. Human liposarcoma cell line SW872 and human osteosarcoma cell line MG63 were treated with ALA in the dark (dark room) for 48 h. (**A**) mRNA expression of iron-metabolism-related genes detected by RT-PCR. The right panel shows the semi-quantification of the RT-PCR signals. (**B**,**C**) The effect of ZnPP on sphere formation in ALA-treated and untreated sarcoma cells. (**D**,**E**) The effect of CoPP on sphere formation in ALA-treated and untreated sarcoma cells. Data in panels, mean ± SD. ALA, 5-aminolevulinic acid; HO-1, heme oxygenase-1; FeCH, ferrochelatase; ZnPP, zinc porphyrin IX; CoPP, cobalt protoporphyrin IX.

To investigate the effect of ALA metabolism on stemness, we examined the expression of ferrochelatase (FeCH) and HO-1, enzymes in the ALA metabolic pathway, under dark conditions (Figure 4A). Our results showed that HO-1 expression increased 1.8–2-fold and FeCH expression increased 2.5–4.7-fold.

To investigate whether HO-1 is involved in the promotion of stemness by ALA, we administered ALA in the dark and simultaneously inhibited HO-1 with ZnPPIX (Figure 4B). Interestingly, in both cell lines, 0.5 mM of ZnPPIX enhanced sphere formation, but 1 mM of ZnPPIX suppressed sphere formation. In contrast, when ALA was not administered, sphere formation was suppressed by ZnPPIX in a concentration-dependent manner (Figure 4C). ALA was then administered in the dark with simultaneous activation of HO-1 and ZnPPIX (Figure 4D), and the results showed that sphere formation decreased in a concentration-dependent manner. In contrast, when ALA was not administered, sphere formation was enhanced by ZnPPIX in a concentration-dependent manner (Figure 4E).

### 2.5. Effects of CO on Stemness in Sarcoma Cells

**Figure 5:**
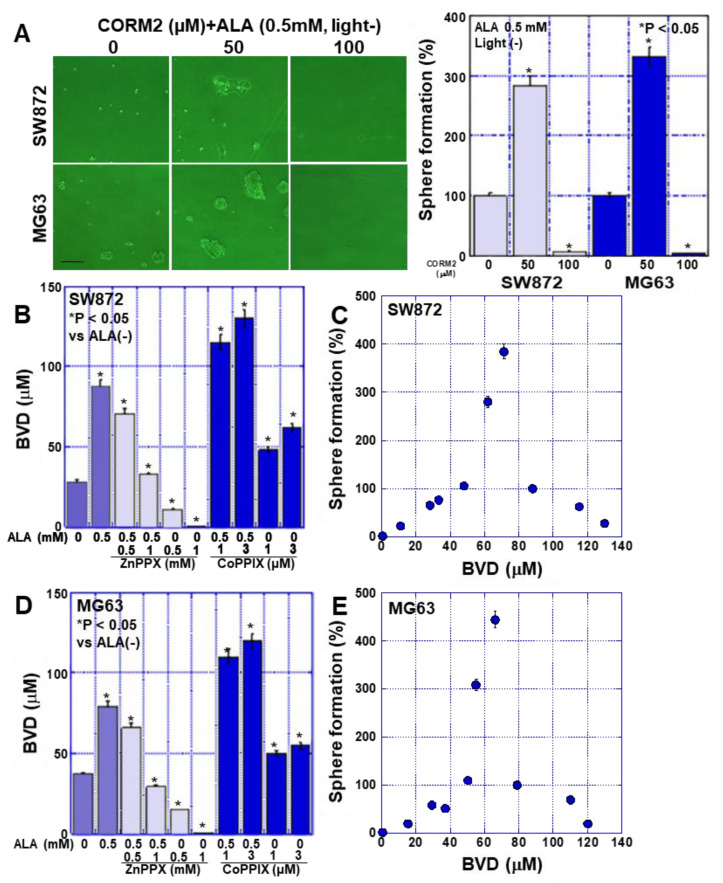
Effect of CORM2 on sphere formation in human sarcoma cell lines. Human liposarcoma SW872 and human osteosarcoma MG63 cells were treated with ALA in the dark (dark room) for 48 h. (**A**) Effect of CORM2 on sphere formation. Scale bar, 100 μm. The right-hand panel shows the number of spheres. (**B**,**D**) The effect of alterations in HO-1 activity on BVD production. (**C**,**E**) Relationship between BVD production and sphere formation. Data in panels, mean ± SD. ALA, 5-aminolevulinic acid; CORM2, carbon monoxide-releasing molecule 2; BVD, biliverdin.

These results suggested that HO-1 affects the stemness of sarcoma cells. HO-1 metabolizes heme to CO, Fe^2+^, and BVD. Therefore, we investigated the effect of CO on stemness (Figure 5). Treatment of sarcoma cells with the CO donor, CORM2, promoted sphere formation at a concentration of 50 mM, whereas sphere formation was suppressed at 100 mM (Figure 5A). This result suggests that CO production by HO-1 affects stemness; therefore, we estimated CO production in previous experiments by measuring the concentration of BVD, a co-product of CO (Figure 5B,D). In both cell types, BVD levels were elevated by ALA, suppressed by ZnPPIX, and enhanced by CoPPIX. Next, the BVD concentration in each experiment was compared with the sphere-forming ability of the cells (Figure 5C,E). Interestingly, sphere formation peaked at a BVD concentration of 60–70 μM, and at higher concentrations of BVD, sphere formation was inhibited in a concentration-dependent manner.

These results suggest that CO produced by HO-1 initially promotes sphere formation but suppresses it at high concentrations. Next, we investigated the mechanism underlying the suppression of sphere formation by high concentrations of ALA.

### 2.6. Effects of High ALA Concentration on Stemness in Sarcoma Cells

**Figure 6:**
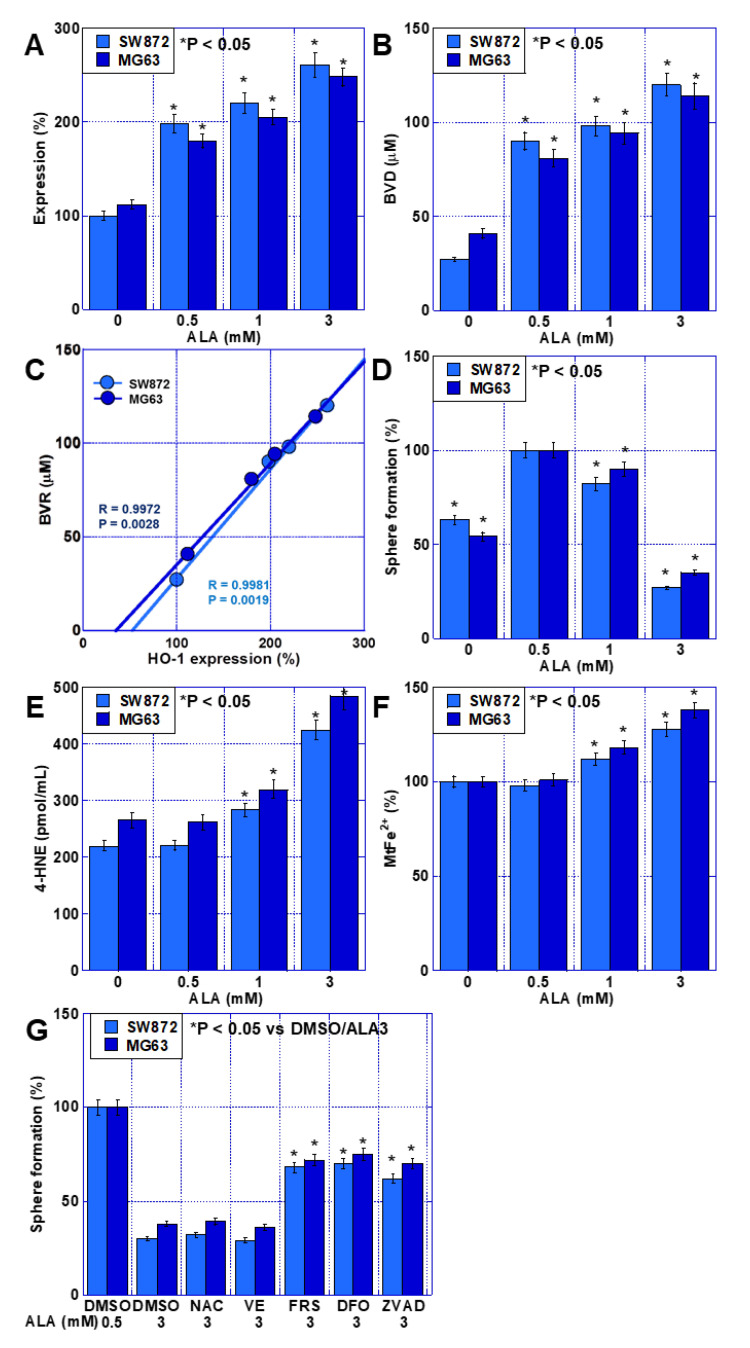
Effect of ALA on cell death in human sarcoma cell lines. Human liposarcoma SW872 and human osteosarcoma MG63 cells were treated with ALA in the dark (dark room) for 48 h. (**A**) Effect of higher ALA dosage (1 and 3 mM) on HO-1 mRNA expression. (**B**) Effect of higher ALA dosage on BVD production. (**C**) Relationship between HO-1 expression and BVD production. The correlation was calculated using Pearson’s R correlation coefficient. (**D**–**F**) Effect of higher ALA dosage on sphere formation (**D**), lipid peroxide formation (4-HNE) (**E**), and mitochondrial Fe^2+^ accumulation (**F**). (**G**) Effect of cell death inhibitors on sphere formation in sarcoma cells treated with ALA (3 mM). Data in panels, mean ± SD. ALA, 5-aminolevulinic acid; BVD, biliverdin; 4-HNE, 4-hydroxynonenal; MtFe^2+^, mitochondrial Fe^2+^; DMSO, dimethyl sulfoxide; NAC, N-acetyl-L-cysteine; VE, vitamin E; FRS, ferrostatin-1; DFO, deferoxamine; ZVAD, Z-VAD-FMK.

When both cell types were treated with 0–3 mM ALA under dark conditions, HO-1 expression increased in a concentration-dependent manner (Figure 6A). BVD concentration also increased in line with ALA concentration, almost parallel to HO-1 expression (Figure 6B). HO-1 expression correlated with the produced BVD concentration (Figure 6C), suggesting that HO-1 expression is proportional to its activity. However, the ability to form spheres was enhanced by 0.5 and 1 mM ALA but suppressed by 3 mM ALA (Figure 6D). In contrast, lipid peroxide (4-HNE) and mitochondrial Fe^2+^ were not increased by 0.5 mM ALA, but they were elevated in response to 1 and 3 mM ALA (Figure 6E,F). We also used various cell death inhibitors to examine the effects of 3 mM ALA on sphere-forming ability (Figure 6G). Our results showed that ferrostatin-1 and deferoxamine (DFO) (both ferroptosis inhibitors), as well as Z-VAD-FMK (an apoptosis inhibitor), rescued the ability of the cells to form spheres to similar levels. In contrast, NAC (water-soluble antioxidant) and VE (lipid-soluble antioxidant) had no rescue effect.

These findings suggest that HO-1 activity correlates with ALA-induced HO-1 expression levels, that 0.5 and 1 mM ALA promote sphere-forming ability, and that 3 mM ALA induces ferroptosis and apoptosis.

## 3. Discussion

When considering systemic administration of 5-ALA, it is important to consider how unexcited 5-ALA acts on tumors in the body, where no light can reach. In this study, we investigated the effects of ALA on sarcoma cells under dark conditions. In the current study, at concentrations reported to be phototoxic in the literature, ALA under dark conditions did not exhibit cytotoxicity but instead promoted stemness. In contrast, high concentrations of ALA suppressed stemness in sarcoma cells under dark conditions. Thus, ALA exhibited contradictory effects on cancer stem cells under dark conditions, depending on its concentration.

In this study, ALA promoted stemness in sarcoma cells, which we believe is caused by ALA-induced HO-1 upregulation and the accompanying CO production. We used the inhibitory derivative ZnPPIX and the stimulatory derivative CoPPIX of FePPIX to modulate the activity of HO-1 [9]. HO-1 expression correlates with HO-1 activity, with ZnPPIX decreasing HO-1 expression and activity and increasing that of CoPPIX [9]. We also confirmed that the induction of HO-1 expression by ALA enhanced HO-1 activity. HO-1 degrades heme to CO, BVD, and Fe^2+^. We found that treatment of sarcoma cells with the CO donor CORM2 enhanced their ability to form spheres. CO enhances stemness and reduces anticancer drug sensitivity and oxidative stress toxicity in cancer cells [19]. Our data showed ALA-induced expression of Oct3 and NS, common stem-cell-associated genes. The Notch and Wnt signaling pathways are involved in mediating these CO-induced effects [20,21]. Our experiments also confirmed the activation of Notch and Wnt by ALA. In contrast, endothelin-1 has been implicated in HO-1-induced enhancements in stemness in colorectal cancer [22]; however, in our study, the expression of endothelin-converting enzyme associated with ALA did not change. These findings suggest that under dark conditions, ALA promotes stemness by activating Notch and Wnt through CO production via HO-1 activation.

Interestingly, our results indicate that excessive HO-1 activation suppresses stemness, in contrast to enhancements in stemness observed at normal ALA concentrations. In the presence of ALA, HO-1 inhibition by ZnPPIX promoted the formation of spheres at low concentrations but suppressed it at high concentrations. In contrast, activation of HO-1 by CoPPIX inhibited sphere formation in the presence of ALA but enhanced it in the absence of ALA. When these apparently contradictory results were examined in terms of sphere-forming ability in relation to the concentration of BVD (a co-product of CO generation by HO-1), a curve with a peak at BVD concentrations of 60–80 μM was found for both sarcoma cell types. Sphere formation decreased at concentrations both lower and higher than those at the peak. This is the first time that this relationship has been reported for these molecules. HO-1 is known to have a cytoprotective effect against oxidative stress and suppressive effect on necrosis, apoptosis, and ferroptosis [23].

We found that HO-1 has a dual role in promoting stemness and inducing cell death. The involvement of the above-mentioned stem-cell-related signals suggests that HO-1 may promote stemness. However, HO-1 and/or its metabolites are also thought to be involved in the suppression of stemness by inducing ferroptosis [8,24]. Since cancer stem cells have higher iron concentrations than non-stem cells, induction of ferroptosis is considered an effective therapeutic tool against cancer stem cells [25]. Our data showed that under dark conditions, 3 mM ALA increased Fe^2+^ retention and lipid peroxide, indicating an anti-stem cell effect, as these factors promote ferroptosis. Furthermore, a cell death inhibitor assay revealed partial rescue by ferrostatin-1 and DFO, suggesting that ferroptosis is involved in the HO-1-mediated cytotoxicity induced by high-concentration ALA without light. HO-1 plays a role in both iron and oxidative stress homeostasis [26,27], and iron overload promotes ROS generation via the Fenton reaction [28], which induces ferroptosis via lipid peroxidation [29,30,31,32]. Increased HO-1 expression promotes heme degradation and ferritin synthesis, alters intracellular iron distribution, and promotes oxidative stress [33,34]. Our data also showed that ALA increased HO-1 expression, mitochondrial Fe^2+^, and lipid peroxidation. However, the results from our cell death inhibitor assay also implicated apoptosis in the suppression of stemness by high concentrations of ALA. This is likely due to the cytotoxicity of CO. Indeed, CO has been shown to exhibit a biphasic effect, being cytotoxic at high concentrations and cytoprotective at low concentrations [35]. The cytotoxicity of CO has been reported in many neuronal cells, and its ability to induce apoptosis has been observed [36,37,38]. Through NF-κB, CO suppresses the expression of anti-apoptotic proteins and induces apoptosis [26,39]. Thus, while excessive activation of HO-1 by high ALA concentrations leads to ferroptosis, simultaneously, CO, a metabolite of HO-1, induces apoptosis. Both of these cell death processes are expected to suppress cancer stemness.

Biliverdin, another heme metabolite produced by HO-1, is not associated with the cytotoxicity of HO-1 due to CO [24]. However, BVD has been reported to induce apoptosis in cancer cells at high concentrations [40]. It is possible that BVD produced alongside CO by HO-1 plays a role in sarcoma cell death. Further detailed investigations to explore this possibility are necessary in the future.

The results of this study showed that HO-1 has dual effects on the induction and suppression of stemness in sarcoma cells, depending on its expression level. With this duality in mind, it is worth noting that the role of HO-1 in ferroptosis was recently re-proposed, as research has demonstrated that it plays both protective and detrimental roles [24,41,42,43]. Moderate levels of HO-1 activation exert cytoprotective effects, whereas overactivation of HO-1 leads to cytotoxicity owing to an excessive increase in labile Fe^2+^ beyond the buffering capacity of ferritin [44,45]. Furthermore, the cytoprotective or apoptosis-inducing effects of the heme metabolites CO and BVD are also determined by their intracellular levels [8]. These findings are in good agreement with our data.

In our study, ALA induced the expression of the iron-metabolizing enzymes FeCH and HO-1. NF-E2-related factor 2 (Nrf2), mitogen-activated protein kinase (MAPK), and heme are known to be involved in HO-1 expression [46]. ALA is believed to promote HO-1 expression through nrf2 and MAPK [47,48], and this pathway was also thought to induce HO-1 expression in this study. In contrast, FeCH converts PPIX to heme, and its induction promotes the supply of substrates for HO-1. FeCH is also a target gene of nrf2 [49], and the induction of its expression by ALA is also thought to be via nrf2. Thus, ALA promotes the FeCH and HO-1 metabolic pathway via nrf2 signaling, resulting in enhanced CO production.

In our experiments, daylight lamps inhibited proliferation and increased oxidative stress following ALA administration, suggesting that PPIX was stimulated. The daylight lamp used in this study had a wavelength of 400–800 nm, which covers the excitation wavelength of PPIX (410 nm). However, because this wavelength range is fairly broad, the excitation level of PPIX is expected to be low. However, daylight lamps have been reported to induce phototoxicity with 5-ALA comparable to sunlight, which may be useful in the treatment of skin tumors [50]. This conversely suggests that exposure to daylight lamps during systemic administration of ALA may cause tissue damage other than that intended for ALA use during surgery [51].

Our data demonstrated the duality of HO-1 expression in malignant cells. This suggests the possibility of enhancing stemness and increasing malignant phenotypes of cancer cells. However, our findings also indicate that the administration of high doses of 5-ALA targeting metastatic lesions may have antitumor effects. Previously, it has been reported that enhanced HO-1 expression promotes ferroptosis by promoting iron accumulation and ROS production through synergistic action with anticancer drugs [41,45]. However, our study lacks in vivo data to discuss this point in detail, and future studies are needed. Although HO-1 has attracted attention as a therapeutic target in cancer [10,52], there is a lack of focus on appropriate HO-1 activity, considering the duality of HO-1 activity that we have reported here. Targeting is expected to lead to more effective antitumor effects. Furthermore, targeting HO-1 using ALA suggests the possibility of developing a new molecular-targeted therapy for sarcomas with poor chemotherapy options.

## 4. Materials and Methods

### 4.1. Cell Culture

The human osteosarcoma cell line MG63 and human liposarcoma cell line SW872 were obtained from the American Type Culture Collection (ATCC; Rockville, MD, USA). Cells were maintained in Dulbecco’s modified Eagle’s medium (DMEM; Sigma-Aldrich Inc., St. Louis, MO, USA) containing 4500 mg/L glucose and 10% fetal bovine serum (Sigma) in a 5% CO_2_ atmosphere at 37 °C.

### 4.2. Reagents

ALA (SBI Pharmaceuticals Co., Ltd., Tokyo, Japan), N-acetyl-L-cysteine (NAC 1 mM) (Sigma-Aldrich Inc., St. Louis, MO, USA), vitamin E (10 μM), dimethyl sulfoxide (DMSO) (WAKO Chemicals, Osaka, Japan), Z-VAD-FMK (ZVAD, 10 μM) (Santa Cruz Biotechnology, Santa Cruz, CA, USA), ferrostatin-1 (FRS, 1 μM), deferoxamine (DFO, 100 μM) (Cayman Chemicals, Ann Arbor, MI, USA), ZnPPIX and CoPPIX (Sigma), and CORM2 (WAKO) were purchased from the manufacturers listed.

### 4.3. Cell Proliferation

Cell proliferation was determined by MTS [3-(4,5-dimethylthiazol-2-yl)-5-(3-carboxymethoxyphenyl)-2-(4-sulfophenyl)-2H-tetrazolium] assay. MTS assays were performed using the Celltiter 96 Aqueous One Solution Cell Proliferation Assay kit (Promega Biosciences, Inc., San Louis Obispo, CA, USA), according to the manufacturer’s instructions.

### 4.4. Mitochondrial Imaging

Mitochondrial functions were examined using fluorescent probes. After treatment with ALA (0.5 mM) under light (daylight lamp) or dark (dark room) conditions, cells were incubated with the probes for 30 min at 37 °C and then photographed using an all-in-one fluorescence microscope (KEYENCE, Osaka, Japan). We used dihydrorhodamine 123 (DHR) (10 μM, Sigma-Aldrich) and Liperfluo (mitochondrial lipid peroxide) (1 μM, Dojindo, Kumamoto, Japan) to assess oxidative stress, MitoGreen (100 nM, PromoCell GmbH, Heidelberg, Germany) to assess mitochondrial volume, tetrathylrhodamine ethyl ester (TMRE) (200 nM, Sigma-Aldrich) to assess MMP, and Mitoferrogreen (20 nM, Dojindo, Kumamoto, Japan) to assess mitochondrial iron (Fe^2+^). Fluorescence images were captured using BZ-X710 (KEYENCE, Osaka, Japan). The captured images were analyzed on a computer using ImageJ software (version 1.52; NIH, Bethesda, MD, USA).

### 4.5. Sphere Formation Assay

Cells (1000 cells/well) were seeded onto uncoated bacteriological 35 mm dishes (Corning Inc., Corning, NY, USA) in 3D Tumorsphere Medium XF (Sigma) [53]. After 7 days of culture, images of the spheres were acquired using an inverted microscope coupled with a camera (Carl Zeiss, Göttingen, Germany). The captured images were analyzed using a computer, and the number of spheres was counted using ImageJ software (version 1.52; NIH, Bethesda, MD, USA).

### 4.6. RNA Isolation

Total cellular RNA was isolated from each sample using TRIzol reagent (Invitrogen, Waltham, MA, USA) and purified using the RNeasy mini kit (Qiagen, Hilden, Germany) according to the manufacturer’s protocol. Purified RNA was quantitated using a NanoDrop ND-1000 spectrophotometer (Thermo Fisher Scientific, Tokyo, Japan).

### 4.7. Reverse-Transcription-Polymerase Chain Reaction (RT-PCR)

Total RNA (1 μg) was used to synthesize cDNA using the ReverTra Ace quantitative PCR (qPCR) RT kit (Toyobo, Osaka, Japan). The PCR reactions were performed according to the manufacturer’s instructions. PCR products were electrophoresed on 2% agarose gels and visualized using ethidium bromide. The primer sets used are listed in Table 1. Primers were synthesized by Sigma-Aldrich (Ishikari, Japan).

### 4.8. Enzyme-Linked Immunosorbent Assay (ELISA)

Whole cell lysates were prepared as previously described using RIPA buffer containing 0.1% SDS (Thermo Fisher Scientific) [54]. Protein assays were performed using the Protein Assay Rapid Kit (Wako Pure Chemical Corporation, Osaka, Japan). Using the extracted proteins, an ELISA kit was used to measure the concentration of 4-hydroxynonenal (HNE) (Abcam, Cambridge, MA, USA) in whole cell lysates according to the manufacturer’s instructions.

### 4.9. Quantification of BVD

We quantified BVD according to the method by Suzuki Y [55]. Diazonium salt solution was prepared as follows: 10 mL of 5 mM 4-chloroanilin in 0.176 mM HCl was added with 72 mM NaNO_2_. The mixture was allowed to stand at room temperature for 120 min until diazotization was complete. Mixture of 0.5 mL of sample solution, 4.5 mL methanol, and the diazonium salt solution was measured for absorbance at 540 nm via a UV-visible spectrophotometer (UV-1280, Shimazu, Kyoto, Japan). A standard curve used for quantification was prepared by measuring a BVD standard sample (WAKO) in the same manner as described above.

### 4.10. Statistical Analysis

Statistical significance was assessed using the Student’s *t*-test with the assumption of a Gaussian distribution according to the Kolmogorov and Smirnov method. The correlation was calculated using Pearson R. All analyses were performed using InStat software (version 3.0, GraphPad, Los Angeles, CA, USA). Statistical significance was defined as *p* < 0.05.

## Figures and Tables

**Table 1:** Primer sets.

Gene	ID		Sequence
*ACTB*	NM_001101.3	Upper	GGACTTCGAGCAAGAGATGG
		Lower	AGCACTGTGTTGGCGTACAG
*HO-1*	NM_002133.2	Upper	TAAGCTGGTGATGGCTTCCT
		Lower	ATGATTTCCTGCCAGTGAGG
*FeCH*	KR712044.1	Upper	GATGAATTGTCCCCCAACAC
		Lower	GCTTCCGTCCCACTTGATTA
*NS*	BC001024.2	Upper	ATTGCCAACAGTGGTGTTCA
		Lower	AATGGCTTTGCTGCAAGTTT
*Oct3*	BC117437.1	Upper	GAAGGATGTGGTCCGAGTGT
		Lower	GTGAAGTGAGGGCTCCCATA
*Wnt1*	NM_005430.4	Upper	CGGCGTTTATCTTCGCTATC
		Lower	GCCTCGTTGTTGTGAAGGTT
*Notch1*	CR457221.1	Upper	GATGTGTGGACTGTGGCACT
		Lower	TGTGTTGCTGGAGCATCTTC
*ECE*	NM_001397.3	Upper	GACAGATGCCTGCTCAACAA
		Lower	GCCCAGGTTGTTTTCTGTGT
*c-MYC*	NM_002467.4	Upper	TTCGGGTAGTGGAAAACCAG
		Lower	CAGCAGCTCGAATTTCTTCC
*PGC-1α*	BC156323.1	Upper	GTGAAGACCAGCCTCTTTGC
		Lower	AATCCGTCTTCATCCACAGG

ACTB, β-actin; HO-1, heme oxygenase-1; FeCH, ferrochelatase; NS, nucleostemin; Oct3, octamer-binding transcription factor-3; ECE, endothelin-converting enzyme; PGC, peroxisome proliferator-activated receptor-gamma coactivator.

## Data Availability

Not applicable.

## Abbreviations

ALA, 5-aminolevulinic acid; PPIX, protoporphyrin; HO-1, heme oxygenase 1; CO, carbon monoxide; CORM2, carbon monoxide-releasing molecule 2; BVD, biliverdin; PGC, proliferator-activated receptor-gamma coactivator; NS, nucleostemin; ECE, endothelin-converting enzyme; HNE, hydroxynonenal; DFO, deferoxamine; FeCH, ferrochelatase; Nrf2, NF-E2-related factor 2.
